# Programmable Auxeticity in Hydrogel Metamaterials via Shape‐Morphing Unit Cells

**DOI:** 10.1002/advs.202201867

**Published:** 2022-06-24

**Authors:** Oliver Skarsetz, Viacheslav Slesarenko, Andreas Walther

**Affiliations:** ^1^ A^3^BMS Lab – Active Adaptive and Autonomous Bioinspired Materials Department of Chemistry Johannes Gutenberg University Mainz Duesbergweg 10–14 Mainz 55128 Germany; ^2^ Cluster of Excellence livMatS @ FIT — Freiburg Center for Interactive Materials and Bioinspired Technologies University of Freiburg Georges‐Köhler‐Allee 105 Freiburg im Breisgau 79110 Germany

**Keywords:** auxetics, hydrogel metamaterials, mechanical metamaterials, shape morphing

## Abstract

Mechanical metamaterials recruit unique mechanical behavior that is unavailable in bulk materials from a periodic unit cell structure with a specific geometry. However, such metamaterials can typically not be reconfigured once manufactured. Herein, the authors introduce shape morphing of a hydrogel metamaterial via spatio‐selective integration of responsive actuating elements to reconfigure the mesoscale unit cell geometry to reach programmable auxeticity on the macroscale. Via thermal control, the unit cell angle of a honeycomb structure can be precisely programmed from 68° to 107°. This results in negative, zero, or positive Poisson's ratio under applied tensile strain. The geometrical reconfiguration with resulting programmable auxeticity is predicted and verified by finite element (FE) simulation. This concept of shape‐morphing hydrogel metamaterials via the addition of actuating struts into otherwise passive architectures offers a new strategy for reconfigurable metamaterials and extends applications of hydrogels in general. It can be readily extended to other architectures and may find applications in mechanical computing as well as soft robotics.

## Graphical abstract

Reconfiguration of the unit cell topology of a hydrogel metamaterial can be achieved by contraction of actuating struts connected to a passive unit cell lattice. This reconfigures the tensile mechanical behavior and enables the materials to switch the Poisson's ratio from positive over zero to negative values. Finite element simulations predict and verify the experimental swelling and subsequent deformation.

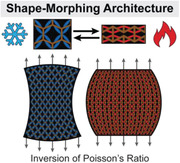

## 1 Introduction

Mechanical metamaterials are artificial materials that obtain unprecedent responses to mechanical forces due to their specifically designed, usually periodic, architectures. Properties such as ultrahigh stiffness,^[^ ^1^, ^2^ ^]^ negative swelling,^[^ ^3^, ^4^, ^5^ ^]^ and negative Poisson's ratio^[^ ^6^, ^7^, ^8^, ^9^ ^]^ have been shown to be achievable through rational structural design. Previous studies have shown that the sign and magnitude of Poisson's ratio can be programmed via geometrical parameters (e.g., angle) of the unit cell.^[^ ^10^, ^11^, ^12^ ^]^ Negative Poisson's ratio is obtained with a re‐entrant unit cell structure with an angle lower than 90°, where re‐entrant struts open up during tensile deformation. This behavior is inverted for angles greater than 90°, resulting in a positive Poisson's ratio where the struts come together during tensile deformation. Most of these carefully designed architectured materials consist of a single material which however imprints the final mechanical behavior. Structural reprogramming of mechanical behavior post manufacturing has been achieved through tensile or compressive deformation above *T*_g_ with subsequent fixation of the internal architecture.^[^ ^13^, ^14^, ^15^ ^]^

Inspiration for reconfigurable metamaterials can be drawn from the field of soft robotics. Soft robotic systems typically contain responsive elements that respond to external stimuli such as heat,^[^ ^16^, ^17^, ^18^, ^19^ ^]^ pH,^[^ ^20^, ^21^, ^22^ ^]^ light,^[^ ^23^, ^24^, ^25^ ^]^ or magnetic fields.^[^ ^26^, ^27^, ^28^ ^]^ To achieve reversible and nonisotropic actuation from isotropic stimuli, responsive, and nonresponsive materials have to be assembled with an asymmetry.^[^ ^29^, ^30^ ^]^ For instance, bilayers composed of responsive and nonresponsive components show bending or twisting motion and are often used in soft robotics.^[^ ^31^, ^32^, ^33^, ^34^, ^35^ ^]^ Encoding such layers into metamaterial architectures allowed to realize tunable stress–strain curves,^[^ ^3^ ^]^ and self‐folding kirigami machines that are able to grip and crawl.^[^ ^36^ ^]^ Besides, reconfiguration of metamaterials was achieved by releasing stress stored in filaments during metamaterial 3D printing.^[^ ^37^ ^]^ However, such a shape reconfiguration with change in Poisson's ratio is unidirectional and nonreversible. Notably, switchable auxetic bulk materials have been fabricated through direct ink writing of functionally graded liquid crystal elastomers (LCE). Through distribution of actuating with nonactuating LCE struts to form a continuous unit cell lattice, the Poisson's ratio of the printed structure under tensile deformation could be switched from negative to positive when the surrounding temperature was increased from room temperature to 100 °C.^[^ ^38^ ^]^ However, general rules in the structural design of auxetic metamaterials to achieve an inversion in Poisson's ratio are not deduced and further insights on the angle‐dependence of Poisson's ratio were not discussed, and remain to be elucidated.

Here, we introduce a fully hydrogel‐based approach for shape‐morphing mechanical metamaterials by combining thermoresponsive actuating hydrogel struts with an otherwise passive re‐entrant unit cell structure that forms a continuous lattice of one material. The actuators exhibit forces onto their elastic surrounding, and hence change the unit cell angle. The use of passive and active hydrogels is critical as the sole use of active materials in the whole geometry would just lead to an isotropic shrinkage with a constant unit cell angle. We will show how the honeycomb structure with angle larger than 90° can be reconfigured into a re‐entrant honeycomb structure with angle smaller than 90°, and that intermediate angles can be programmed through careful thermal control. Furthermore, this architectural shape morphing results in different responses to applied mechanical forces, that is, the resulting Poisson's ratio can be tuned from negative over zero to positive values through postfabrication alteration of the structural angle.

## 2 Results and Discussion

**Scheme 1:**
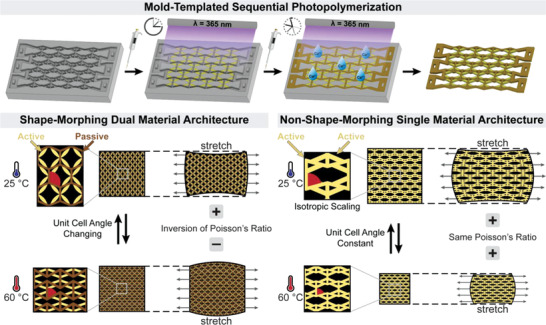
Schematic illustration of mold‐templated sequential photopolymerization and the resulting shape‐morphing and nonshape‐morphing architecture at different thermal equilibria. The unit cell structure is actuated from conventional honeycomb to re‐entrant auxetic structure with change in unit cell angle resulting in an inversion of Poisson's ratio. In contrast, the non‐shape‐morphing architecture consisting of a single active material keeps its unit cell structure at different thermal equilibria. The unit cell angle and resulting Poisson's ratio remain constant while the whole structure scales isotropically.

In more detail, our soft shape‐morphing architecture is composed of two different hydrogels. A passive (i.e., non‐thermoresponsive) hydrogel that forms a continuous lattice is combined with active (i.e., thermoresponsive) hydrogel connectors that serve as actuating struts. The passive hydrogel maintains constant volume while the swelling of the active hydrogel can be altered by temperature. These active hydrogel actuating struts are incorporated into a re‐entrant honeycomb architecture with a starting angle of 75° (**Scheme** **1**).

**Figure 1:**
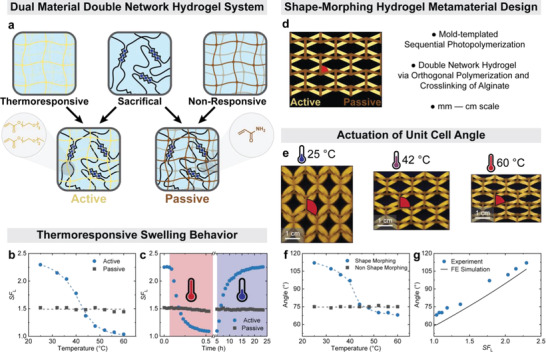
Characterization of the dual material double network hydrogel system and the shape‐morphing architecture. a) The active double network hydrogel consists of thermoresponsive poly(mTEGA_60_‐_co_‐eDEGA_40_) loosely crosslinked with 0.03 mol% Bis‐AAm, together with 1.56 wt% of alginate ionically crosslinked with 0.2 m CaCl_2_. The passive double network hydrogel consists of nonresponsive acrylamide loosely crosslinked with 0.03 mol% Bis‐AAm, together with 1.56 wt% of alginate ionically crosslinked with 0.2 m CaCl_2_. b) The *SF*_L_ of the reference rod‐like active and passive hydrogel specimens after equilibration in water at different temperatures. c) Actuation of the active and passive hydrogels: Hydrogels swollen to equilibrium at room temperature are exposed to water at 60 °C while the length *SF*_L_ = *L*/*L*_dry_ is measured over time. After 5 h, the high temperature stimulus is removed. The active hydrogel shows time‐dependent contraction at high temperature and reswelling at low temperature. d) Scheme of the shape‐morphing hydrogel metamaterial geometry with the respective active and passive hydrogel with a synthesized angle of 75°. e) The respective shape‐morphing structure after equilibrating at different temperatures: The structural angle is 107°, 90°, and 68° at 25, 42, and 60 °C, respectively. f) The structural angle is determined for the shape‐morphing and passive non‐shape‐morphing geometry after equilibration at different temperatures. g) The structural unit cell angle of the shape‐morphing geometry against the *SF*_L_ of the reference rod‐like active specimen in comparison to the FE simulations.

The hydrogels were fabricated via sequential photopolymerization using PTFE molds by first polymerizing the active struts, followed by polymerization of the passive part. The connection points of active struts with passive architecture are predefined in the mold (Scheme 1). During the sequential photopolymerization, the precursor solution for the passive hydrogels diffuses to some extent into the already polymerized active struts, resulting in good cohesion between the active and passive parts. These tight connections allow the shape‐morphing architecture to be repeatedly actuated and repeatedly deformed without structural rupture. To increase overall mechanical robustness and crack resistance of the composite metamaterial hydrogels, we opted for a double network structure with Ca^2+^‐crosslinked alginate (1.56 wt%) in both active struts and passive architecture. This means that the photo‐crosslinked structures were subsequently crosslinked in a 0.2 m CaCl_2_ solution. The nonresponsive, passive hydrogel is composed of nonresponsive acrylamide (AAm) loosely crosslinked with 0.03 mol% *N*‐*N*′‐methylene bisacrylamide (Bis‐AAm) (**Figure** **1**a). AAm is selected as a monomer for the passive hydrogel due to its nonresponsiveness, and because it keeps its size after preparation without additional swelling. The responsive, active hydrogel parts consist of a copolymer of triethylene glycol methyl ether acrylate (mTEGA), diethylene glycol ethyl ether acrylate (eDEGA), and Bis‐AAm crosslinker with the following molar ratios (mTEGA:eDEGA:Bis‐AAm = 60:40:0.03). This copolymer composition was selected due to the tunability of the solubility‐to‐insolubility transition; here with a cloud point of 47 °C.^[^ ^39^ ^]^ Beneficially, a higher *SF*_L_ compared to the commonly used poly(*N*‐isopropylacrylamide) can be achieved too (Figure S2a, Supporting Information). The double network hydrogels possess Young's moduli of *E*_active_ = 7.5 kPa and *E*_passive_ = 75 kPa after preparation, respectively (Figure S3a, Supporting Information).

After fabrication, the composite hydrogels are placed into water to allow for equilibrium swelling, which is material‐dependent and hence different for both active and passive gel parts. During swelling at room temperature, the angle reconfigures from 75° of the mold to 107° in water. This can be understood by comparing to swelling tests conducted on individual gel rods (not attached to any geometry) of both materials. The swelling factor of freely swollen versus fully dry, *SF*_L_ = *L*/*L*_dry_ (*L* = length), of an active reference gel is *SF*_L__, active_ = 2.26, while that of a passive one is *SF*_L__, passive_ = 1.52 at room temperature, respectively. The initial swelling ratio for both after preparation in the mold is *SF*_L__, prepared_ = 1.48. Hence a material‐selective swelling takes place reconfiguring the angle of the molded structure to the freely water swollen structure.

When slowly increasing the temperature in 4 °C increments with subsequent two‐hour equilibration, the freely swollen reference active hydrogel specimen decreases in size from *SF*_L_ = 2.26 at room temperature to *SF*_L_ = 1.04 at 60 °C (Figure 1b). This very low *SF*_L_ corresponds to an almost complete removal of water from the hydrogel. The strongest decrease occurs around the volume phase transition temperature (VPTT) of ≈ 47 °C. In contrast, the freely swollen reference passive hydrogel specimen remains at constant size of *SF*_L_ ≈ 1.5. Furthermore, when the active hydrogels are directly placed into a 60 °C water bath, a similar, total deswelling (i.e., collapse) to *SF*_L_ = 1.09 with an actuation time of ≈ 27 min takes place, while reswelling at room temperature takes place over 18 h (Figure 1c).

These differences in the temperature‐dependent volume transitions of both hydrogels trigger shape morphing of the composite hydrogel unit cell when changing the temperature (Figure 1d). An increase of the temperature from room temperature to 42 °C changes the initial angle of 105° to 90°, while equilibration at 60 °C leads to an angle of 68° (Figure 1e,f). A comparison of the change in structural angle of the shape‐morphing metamaterial hydrogel with the collapse of a small rod‐like reference specimen of pure active material (from Figure 1b,f) reveals a direct correlation of the hydrogel collapse with the geometrical reconfiguration of the unit cell (Figure 1g). Corresponding finite element (FE) simulations, which use the experimentally determined Young's moduli at 25 °C and *SF*_L_ of active and passive material as input (see FE simulations in the Supporting Information), further confirm this linear trend: An increase in active strut size leads to an increase in unit cell angle. In contrast, the angle of a non‐shape‐morphing reference specimen, which was fabricated using only the active or passive hydrogel, remains constant at the synthesis angle of around 75° after equilibration at 25–60 °C (Figure 1f; Figure S4, Supporting Information). This data clearly shows that specific temperatures can reconfigure the unit cell angle from significantly larger than 90° to significantly smaller than 90° in a programmable manner after manufacturing of the metamaterials.

**Figure 2:**
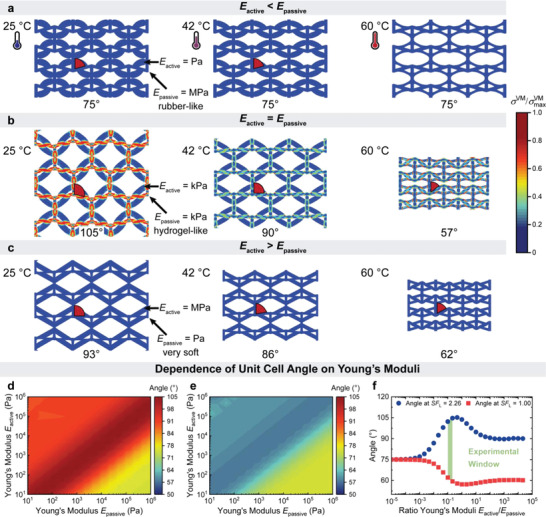
FE simulations of the shape‐morphing architecture. The active struts of the shape‐morphing architecture are fully swollen (*SF*_L_ = *L*/*L*_dry_ = 2.26), partly swollen (*SF*_L_ = 1.85) and contracted (*SF*_L_ = 1.04) with experimentally obtained *SF*_L_. Resulting geometries after swelling to three equilibria are depicted. Maximum von Mises stress *σ* ^VM^_max_ = 40 kPa. a) The active hydrogel is very soft (100 Pa) while the passive architecture is stiff (1 MPa). All structures have a unit cell angle of 75°. b) The active hydrogel has the same stiffness (75 kPa) as the passive architecture. The obtained structures have a unit cell angle of 105°, 90°, and 57° in the swollen, partly swollen, and contracted state. c) The active hydrogel has a high stiffness (1 MPa) while the passive is soft (100 Pa). The obtained structures have unit cell angles of 93°, 86°, and 62° in the swollen, partly swollen, and contracted state. d) Contour plot of the angle in swelling equilibrium (*SF*_L_ = 2.26) at different Young's moduli of active struts and passive architecture. e) Contour plot of the angle in contraction equilibrium (*SF*_L_ = 1.04) at different Young's moduli of active struts and passive architecture. f) Maximum (*SF*_L_ = 2.26) and minimum (*SF*_L_ = 1.04) swelling angle for different Young's moduli ratio *E*_active_/*E*_passive_. The window of experimentally employed Young's moduli ratio is highlighted in green.

For the reconfiguration of the angle to work, it is important to realize that the active hydrogel struts in fact work as small actuating muscles on the passive gel lattice. Although this intuitively requires balanced mechanical properties, details on the influence of a mismatch of the mechanical properties are unknown. To address this challenge, we next rationalize the extent of shape morphing of the angle during simulated swelling and collapse using FE simulations for different material stiffnesses (Young's moduli) of active and passive materials. To this end, we used experimental swelling data of the active material at room temperature and 60 °C as an input for the simulated swelling (**Figure** **2**). The starting angle and geometrical dimensions were set to match the experiment. The Young's moduli were varied from 100 to 1 MPa ( = 10^6^ Pa) to cover a material range from extremely soft hydrogels to rubber‐like materials.

Figure 2a–c depicts three examples for extreme combinations of moduli. The color scale corresponds to von Mises stress, which represents the entire stress tensor as a single value at any point in the geometry. Generally speaking, this can be treated as a measure of stress intensity. (a) For very soft active struts (100 Pa) and a stiff passive architecture (1 MPa), the structural angle remains constant at 75°, no matter whether the active part is collapsed or swollen. This can be rationalized by simply considering that the soft hydrogel cannot deform a much stiffer passive grid (Figure 2a). (b) For equal Young's moduli of active struts and passive architecture, the resulting angle increases to 105° when fully swollen and decreases to 57° when fully collapsed (Figure 2b). Note that the active and passive hydrogels possess a Young's moduli ratio of *E*_active_/*E*_passive_ = 0.1 (Figure S3a, Supporting Information). (c) For stiff active struts (1 MPa) and a very soft passive lattice (100 Pa), the structural angle reaches 90° when fully swollen and decreases to 62° when fully contracted (Figure 2c). Furthermore, the resulting maximum von Mises stress is also greatest with equal Young's moduli (*σ* ^VM^_max_ = 40 kPa) compared to the stiff (*σ* ^VM^_max_ = 0.12 kPa) or very soft active struts (*σ* ^VM^_max_ = 1 kPa), which further proves that greatest shape morphing is achieved when active and passive materials align mechanically, and synergistically allow the transfer of forces during swelling.

To further quantify the behavior, Figure 2d,e displays the contour plots of the angle in swollen and collapsed state, respectively. The largest angle in swelling conditions is found where *E*_active_ ≈ *E*_passive_, as seen by the red diagonal (Figure 2d). The respective contour plot of the angle in collapsed state follows the same trend. The minimum angle in collapsed state is also found where *E*_active_ ≈ *E*_passive_, as seen by the blue diagonal (Figure 2e). To closer evaluate this feature, Figure 2f displays the magnitude of possible reconfiguration of the unit cell angle in swollen and collapsed condition against the ratio of both Young's moduli *E*_active_/*E*_passive_. Both angles in swollen and collapsed state are constant at a Young's moduli ratio *E*_active_/*E*_passive _< 10^−3^, which corresponds to soft actuating struts in a stiff unit cell lattice. The maximum changes are reached around *E*_active_ ≈ *E*_passive_. A maximum angle of 105° in swollen state and a minimum angle of 57° in collapsed state is found. A further increase in the Young's moduli ratio towards softer passive structures and stiffer active materials results in a decrease in the maximum angle in swollen conditions (90°), which then plateaus above *E*_active_/*E*_passive_ > 10^2^. The respective minimum angle in collapsed state also reaches a plateau at 60° above *E*_active_/*E*_passive_ > 10^2^.

**Figure 3:**
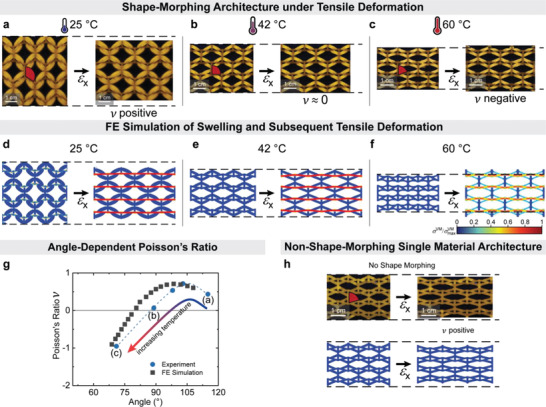
Poisson's ratio of the dual material geometry under room temperature tensile deformation at different swelling equilibria. The shape‐morphing architecture swollen to equilibrium a) at 25 °C shows positive, b) at 42 °C shows zero, and c) at 60 °C shows negative Poisson's ratio at an extension of *ε*_x_ = 10%. FE simulations of the actuation and subsequent deformation of the shape‐morphing geometry at the swelling equilibria corresponding to 25, 42, and 60 °C. d) The swollen (*SF*_L__, active_ = 2.26) geometry shows positive, e) partly swollen (*SF*_L__, active_ = 1.67) geometry shows zero, and f) contracted (*SF*_L__, active_ = 1.04) geometry shows negative Poisson's ratio at *ε*_x_ = 10 %. *σ* ^VM^_max_ = 45 kPa. g) Experimental and simulated Poisson's ratios at *ε*_x_ = 10% versus angle at different equilibrated angles. Specimens depicted in (a)–(c) are indicated next to additional temperatures not shown in (a)–(c). *σ* ^VM^_max_ = 45 kPa. h) Single material geometry with corresponding FE simulation swollen to equilibrium at 25 °C is stretched. In both simulation and experiment, the Poisson's ratio is determined from the change in lateral dimension under tensile deformation in relation to the vertical dimension of the unit cell in the center (see determination of the unit cell angle and Poisson's ratio in the Supporting Information).

Next, we capitalize on the reprogrammability of the unit cell angle and measure the Poisson's ratio of the shape‐morphing metamaterial hydrogel under tensile deformation at different swelling equilibria as obtained from equilibrating at different temperatures. We first focus on three representative experimental situations at an extension of *ε*_x_ = 10%, to which the shape‐morphing architecture can be stretched in all three swelling equilibria without structural rupture (Figure S5a, Supporting Information). When equilibrated at room temperature (25 °C), the metamaterial hydrogel features a unit cell angle of 107°, and consequently shows a positive Poisson's ratio of *ν* = 0.5 during stretching (**Figure** **3**a). The material diminishes its width in the nonstretched directions, as indicated by the dashed lines in Figure 3a. However, after equilibration at 42 °C, the unit cell angle reconfigures to 90°, and accordingly a Poisson's ratio around zero is obtained (Figure 3b). Lastly, after equilibration at 60 °C an angle of 68° is found, that now leads to a negative Poisson's ratio of *ν* = −1 (Figure 3c). The lateral dimensions clearly expand during stretching as again indicated by the dashed lines in Figure 3c.

Additional FE simulations confirm the reprogrammability of Poisson's ratio. A positive Poisson's ratio of *ν* = 0.5 at *ε*_x_ = 10% is found for *SF*_L__, active_ = 2.26 (low temperature; Figure 3d). Furthermore, after collapsing to *SF*_L__, active_ = 1.67, a Poisson's ratio of *ν* = 0 is obtained (intermediate temperature; Figure 3e). Lastly, a further collapse to *SF*_L__, active_ = 1.04 results in a negative Poisson's ratio around *ν* = −1 (high temperature; Figure 3f). To further quantify the dependence of the Poisson's ratio at different temperature/swelling equilibria/angles, Figure 3g compares the Poisson's ratio at *ε*_x_ = 10% for both experiments and simulations. Both show the same trend. The experimentally obtained Poisson's ratios show a transition from negative to positive and a maximum of *ν* = 0.7 is obtained at 105°, which subsequently decreases to *ν* = 0.4 at 115°. The downturn at high unit cell angles may possibly be due to appearance of a bending‐dominated deformation in the active struts after large swelling. The experimentally determined and simulated angles for the Poisson's ratios show a minor offset of ≈10°, which we associate to some unavoidable challenges in modeling the exact mechanical behavior of the polymers that is a function of temperature. Further simulations with different Young's moduli ratio *E*_active_/*E*_passive_ are shown in Figure S5b in the Supporting Information. As a control experiment, the Poisson's ratio of a non‐shape‐morphing geometry was determined under tensile deformation at different temperatures (Figure S6a, Supporting Information). The Poisson's ratio remains constant around *ν* = 0.5 after equilibration at 25 °C (Figure 3h; *SF*_L__, passive_ = 1.52) as well as at 60 °C (*SF*_L__, passive_ = 1.44). FE simulations (Figure 3h; *SF*_L__, passive_ = 1.52) confirm that the positive Poisson's ratio remains unchanged.

## 3 Conclusion

In summary, we introduced the shape morphing of hydrogel metamaterials, in which active actuating struts incorporated into an otherwise passive re‐entrant auxetic structure give structural control over the unit cell angle after manufacturing and in response to external stimulus. The re‐entrant auxetic structure can be thermally actuated from 68°‐unit cell angle to 107° resulting in a honeycomb like structure which also results in the inversion of Poisson's ratio (−/**0**/**+**) under applied tensile strains. The fabrication of a single shape‐morphing specimen that can be reversibly actuated to achieve switchable auxeticity from positive, zero, or negative values removes the need to manufacture three individual geometries for each desired Poisson's ratio, and opens pathways for adaptive mechanical metamaterials with a reversible behavior. Our shape‐morphing geometry shows great system control since temperature defines actuation size which results in specific structural angles further determining the Poisson's ratio. We showed how FE simulations predict the shape morphing of the re‐entrant unit cell structure as well as the Poisson's ratio under tensile deformation. Importantly, we showed how the Young's moduli of active and passive material need to be balanced to allow for large reconfiguration. The shape morphing is greatest around *E*_active_ ≈ *E*_passive_.

Experimentally, we also introduced a new manufacturing strategy for the preparation of shape‐morphing composite hydrogel metamaterials with high structural resolution via mold‐templated sequential photopolymerization, and showed that mechanical properties can be critically enhanced using double network strategies compatible with responsive polymers. Furthermore, our strategy of mold‐assisted sequential photopolymerization to fabricate shape‐morphing hydrogel metamaterials is not limited to two materials or temperature as stimulus. In principle, any number of active and passive hydrogel material can be combined into one geometry with spatial control. Therefore, our strategy can be readily extended to other architectures and hydrogels responding to multiple orthogonal environmental stimuli. This opens up a considerable design space for shape‐morphing hydrogel metamaterials.

Looking at possible applications, stimuli‐responsive hydrogels have been proven to be important in various applications such as soft robotics, tissue engineering and as soft implant materials due to their high compliance and programmability. The use of shape‐morphing hydrogels expands the range of kinematic motions in which geometrical reconfiguration can be harnessed for soft implant materials, whose size and mechanical properties can be programmed after deployment.

## Conflict of Interest

The authors declare no conflict of interest.

## Supporting information

Supporting Information

Supplemental Video 1

## Data Availability

The data that support the findings of this study are available in the supplementary material of this article.

## References

[1] X. Zheng , H. Lee , T. H. Weisgraber , M. Shusteff , J. DeOtte , E. B. Duoss , J. D. Kuntz , M. M. Biener , Q. Ge , J. A. Jackson , S. O. Kucheyev , N. X. Fang , C. M. Spadaccini , Science 2014, 344, 1373. 24948733 10.1126/science.1252291

[2] J. B. Berger , H. N. G. Wadley , R. M. McMeeking , Nature 2017, 543, 533. 28219078 10.1038/nature21075

[3] H. Zhang , X. Guo , J. Wu , D. Fang , Y. Zhang , Sci. Adv. 2018, 4, eaar8535. 29888326 10.1126/sciadv.aar8535 PMC5993477

[4] Z. Chen , Y. Li , Q. M. Li , Mater. Des. 2021, 207, 109819.

[5] J. Liu , T. Gu , S. Shan , S. H. Kang , J. C. Weaver , K. Bertoldi , Adv. Mater. 2016, 28, 6619. 27184443 10.1002/adma.201600812

[6] H. M. A. Kolken , A. A. Zadpoor , RSC Adv. 2017, 7, 5111.

[7] M. J. Mirzaali , S. Janbaz , M. Strano , L. Vergani , A. A. Zadpoor , Sci. Rep. 2018, 8, 965. 29343772 10.1038/s41598-018-19381-3 PMC5772660

[8] F. Wenz , I. Schmidt , A. Leichner , T. Lichti , S. Baumann , H. Andrae , C. Eberl , Adv. Mater. 2021, 33, 2008617. 10.1002/adma.202008617 PMC11469262 34338367

[9] S. Babaee , J. Shim , J. C. Weaver , E. R. Chen , N. Patel , K. Bertoldi , Adv. Mater. 2013, 25, 5044. 23878067 10.1002/adma.201301986

[10] R. Lakes , Science 1987, 235, 1038. 17782252 10.1126/science.235.4792.1038

[11] T. Bückmann , N. Stenger , M. Kadic , J. Kaschke , A. Frölich , T. Kennerknecht , C. Eberl , M. Thiel , M. Wegener , Adv. Mater. 2012, 24, 2710. 22495906 10.1002/adma.201200584

[12] G. N. Greaves , A. L. Greer , R. S. Lakes , T. Rouxel , Nat. Mater. 2011, 10, 823. 22020006 10.1038/nmat3134

[13] M. A. Wagner , T. S. Lumpe , T. Chen , K. Shea , Extreme Mech. Lett. 2019, 29, 100461.

[14] C. Yuan , X. Mu , C. K. Dunn , J. Haidar , T. Wang , H. Jerry Qi , Adv. Funct. Mater. 2018, 28, 1705727.

[15] C. Yang , M. Boorugu , A. Dopp , J. Ren , R. Martin , D. Han , W. Choi , H. Lee , Mater. Horiz. 2019, 6, 1244.

[16] C. A. Spiegel , M. Hippler , A. Münchinger , M. Bastmeyer , C. Barner‐Kowollik , M. Wegener , E. Blasco , Adv. Funct. Mater. 2020, 30, 1907615.

[17] G. Gao , Z. Wang , D. Xu , L. Wang , T. Xu , H. Zhang , J. Chen , J. Fu , ACS Appl. Mater. Interfaces 2018, 10, 41724. 30387979 10.1021/acsami.8b16402

[18] Y. S. Kim , M. Liu , Y. Ishida , Y. Ebina , M. Osada , T. Sasaki , T. Hikima , M. Takata , T. Aida , Nat. Mater. 2015, 14, 1002. 26259107 10.1038/nmat4363

[19] S. Xiao , M. Zhang , X. He , L. Huang , Y. Zhang , B. Ren , M. Zhong , Y. Chang , J. Yang , J. Zheng , ACS Appl. Mater. Interfaces 2018, 10, 21642. 29878750 10.1021/acsami.8b06169

[20] C. Yu , P. Yuan , E. M. Erickson , C. M. Daly , J. A. Rogers , R. G. Nuzzo , Soft Matter 2015, 11, 7953. 26323563 10.1039/c5sm01892g

[21] Z. Han , P. Wang , G. Mao , T. Yin , D. Zhong , B. Yiming , X. Hu , Z. Jia , G. Nian , S. Qu , W. Yang , ACS Appl. Mater. Interfaces 2020, 12, 12010. 32053341 10.1021/acsami.9b21713

[22] S. J. A. Houben , S. J. D. Lugger , R. J. H. van Raak , A. P. H. J. Schenning , ACS Appl. Polym. Mater. 2022, 4, 1298.

[23] C. Li , A. Iscen , L. C. Palmer , G. C. Schatz , S. I. Stupp , J. Am. Chem. Soc. 2020, 142, 8447. 32330027 10.1021/jacs.0c02201

[24] S. Xiang , Y. Su , H. Yin , C. Li , M. Zhu , Nano Energy 2021, 85, 105965.

[25] W. Fan , C. Shan , H. Guo , J. Sang , R. Wang , R. Zheng , K. Sui , Z. Nie , Sci. Adv. 2019, 5, eaav7174. 31016242 10.1126/sciadv.aav7174 PMC6474766

[26] Y. Alapan , A. C. Karacakol , S. N. Guzelhan , I. Isik , M. Sitti , Sci. Adv. 2020, 6, eabc6414. 32948594 10.1126/sciadv.abc6414 PMC7500935

[27] S. M. Montgomery , S. Wu , X. Kuang , C. D. Armstrong , C. Zemelka , Q. Ze , R. Zhang , R. Zhao , H. J. Qi , Adv. Funct. Mater. 2021, 31, 2005319.

[28] V. Slesarenko , Materials (Basel) 2020, 13, 1313. 10.3390/ma13061313 PMC7143886 32183196

[29] Y. Zhao , M. Hua , Y. Yan , S. Wu , Y. Alsaid , X. He , Annu. Rev. Control Robot. Auton. Syst. 2022, 5, 515.

[30] G. Decroly , A. Toncheva , L. Blanc , J.‐M. Raquez , T. Lessinnes , A. Delchambre , P. Lambert , Actuators 2020, 9, 131.

[31] H. Arslan , A. Nojoomi , J. Jeon , K. Yum , Adv. Sci. 2019, 6, 1800703. 10.1002/advs.201800703 PMC6343088 30693178

[32] M. Hippler , E. Blasco , J. Qu , M. Tanaka , C. Barner‐Kowollik , M. Wegener , M. Bastmeyer , Nat. Commun. 2019, 10, 232. 30651553 10.1038/s41467-018-08175-w PMC6335428

[33] L. Ionov , Adv. Funct. Mater. 2013, 23, 4555.

[34] I. Apsite , S. Salehi , L. Ionov , Chem. Rev. 2022, 122, 1349. 34958196 10.1021/acs.chemrev.1c00453

[35] J. Odent , S. Vanderstappen , A. Toncheva , E. Pichon , T. J. Wallin , K. Wang , R. F. Shepherd , P. Dubois , J. M. Raquez , J. Mater. Chem. A 2019, 7, 15395.

[36] Y. Tang , Y. Li , Y. Hong , S. Yang , J. Yin , Proc. Natl. Acad. Sci. USA 2019, 116, 26407. 10.1073/pnas.1906435116 PMC6936366 31843912

[37] T. van Manen , S. Janbaz , K. M. B. Jansen , A. A. Zadpoor , Commun. Mater. 2021, 2, 56.

[38] Z. Wang , Z. Wang , Y. Zheng , Q. He , Y. Wang , S. Cai , Sci. Adv. 2020, 6, eabc0034. 32978149 10.1126/sciadv.abc0034 PMC7518867

[39] L. Voorhaar , K. Van Hecke , G. Vancoillie , Q. Zhang , R. Hoogenboom , in Controll. Radic. Polym. Mater, Vol. 1188 (Eds: K. Matyjaszewski , B. S. Sumerlin , N. V. Tsarevsky , J. Chiefari ), ACS, Washington, DC, USA 2015, Ch. 5.

