# Supplementary material for: Programmable Auxeticity in Hydrogel Metamaterials via Shape‐Morphing Unit Cells
Source: Adv Sci (Weinh). 2022 Jun 24;9(23):2201867. doi: 10.1002/advs.202201867 (PMC9376742; doi:10.1002/advs.202201867)
Supplement: Supplementary file 1 — Supporting Information [file ADVS-9-2201867-s002.pdf]

ADVANCED SCIENCE
Open Access

Supporting Information

for *Adv. Sci.*, DOI 10.1002/advs.202201867

Programmable Auxeticity in Hydrogel Metamaterials via Shape-Morphing Unit Cells

Oliver Skarsetz, Viacheslav Slesarenko and Andreas Walther**

Supporting Information

Programmable Auxeticity in Hydrogel Metamaterials via Shape-Morphing Unit Cells

Oliver Skarsetz, Viacheslav Slesarenko, and Andreas Walther**

O. Skarsetz, A. Walther

A[3]BMS Lab – Active, Adaptive and Autonomous Bioinspired Materials, Department of Chemistry, Johannes Gutenberg University Mainz, Duesbergweg 10-14, 55128 Mainz, Germany

E-Mail: andreas.walther@uni-mainz.de

V. Slesarenko, A. Walther

Cluster of Excellence livMatS @ FIT — Freiburg Center for Interactive Materials and Bioinspired Technologies, University of Freiburg, Georges-Köhler-Allee 105, 79110 Freiburg im Breisgau, Germany

E-Mail: viacheslav.slesarenko@livmats.uni-freiburg.de

1 Experimental Section

Materials: Methylene bis acrylamide (Bis-AAm), acrylamide (AAm), sodium alginate from brown algae (medium viscosity), lithium phenyl-2,4,6,-trimethylbenzoylphosphinate (LAP) ⩾95% were purchased from Sigma Aldrich. Calcium chloride dihydrate ($CaCl_2$) >99% was purchased from Alfa Aesar. Acryloxyethyl thiocarbamoyl Rhodamine B was purchased from Polysciences, Inc. Triethylene glycol methyl ether acrylate (mTEGA) >98% and diethylene glycol ethyl ether acrylate (eDEGA) were purchased from TCI. mTEGA and eDEGA monomers are passed through a short column of basic alumina prior to use to remove the present radical inhibitor. All other chemicals are used as received without further purification.

Hydrogel Precursor Solutions: The precursor are adapted from a previously reported synthesis of J. Y. Sun et al.[39] The **passive double network hydrogel** precursor was prepared as follows: The first network components consisting of 1.75 M AAm crosslinked with 0.03 mol% Bis-AAm and 0.1 wt% photoinitiator LAP is dissolved in water together with the second network component alginate (1.56 wt%). The **active double network hydrogel** precursor is prepared similarly: The first network components consisting of the thermoresponsive monomer combination mTEGA (0.87 M) and eDEGA (0.58 M) with cloud point of 47 °C,[38] crosslinked with 0.03 mol% Bis-AAm and 0.1 wt% photoinitiator LAP is dissolved in water together with the second network component alginate (1.56 wt%). Acryloxyethyl thiocarbamoyl Rhodamine B is added to both hydrogel precursor solutions to enhance the visual contrast.

Manufacturing of PTFE molds: The shape-morphing geometries are prepared in PTFE molds: 2D computer aided design (CAD) models with a depth of 2 mm were milled into PTFE blocks (X*Y*Z = 100*60*10 mm^3) using a computer numerical control (CNC) machine with a 1 mm diameter milling tool. For analysis of the 1D swelling length, rectangular geometries (20*4*2 mm^3) are milled. For the shape-morphing geometry with depth 2 mm, the individual thickness of the passive re-entrant unit cell structure is 1.5 mm with an active strut thickness of 1.84 mm. All the other geometrical dimensions are depicted in Figure S1.

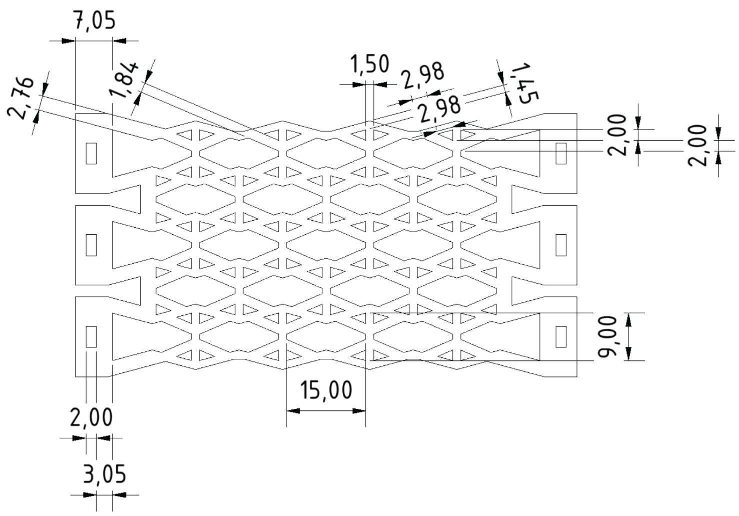

Figure S1: Dimensions in mm of the milled shape-morphing architecture with a starting angle of 75°.

Photopolymerization of the rectangular hydrogel or shape-morphing hydrogel architecture: Hydrogels are manufactured via free radical photopolymerization followed by ionic crosslinking: For single material geometries, the precursor solution is carefully filled into the mold followed by exposure to 60 seconds of 365 nm UV light (four 9 W bulbs, maximum intensity wavelength λ = 365 nm). Afterwards, the whole mold is soaked with 0.2 M $CaCl_2$ solution to ionically crosslink the alginate. After 15 minutes, the specimen is removed from the mold and immersed in more $CaCl_2$ solution to homogeneously form the ionic crosslinks. Two hours later, the $CaCl_2$ solution is removed, and the specimen is placed in an excessive amount of DI water to remove residual $CaCl_2$ and left to equilibrate overnight. The shape-morphing geometry is manufactured likewise with an additional photopolymerization step: First, the active hydrogel precursor solution is filled into the mold then exposed to 15 seconds of UV light (λ = 365 nm). Afterwards, the passive hydrogel precursor solution is filled into the mold followed by UV crosslinking (λ = 365 nm) for 60 seconds. Alginate dissolved inside both active and passive photocrosslinked hydrogels is then ionically crosslinked by soaking the mold with 0.2 M $CaCl_2$ solution. After 15 minutes, the hydrogel geometry is removed from the mold and immersed in more $CaCl_2$ solution. After 2 hours, the geometry is immersed in DI water to equilibrate overnight.

Thermal Actuation of the Thermoresponsive Geometries: The geometries are actuated by immersion into a heat-controlled water bath (VWB2 5) where the hydrogels are immersed for at least 2 hours at a certain temperature. The length of rectangular hydrogels is measured with a caliper. Since hydrogel re-swelling takes place over hours, the hydrogels can be measured outside of the water bath at room temperature without significant dimensional change. This length is then compared to the respective dry length (13.5 mm for both active and passive double network hydrogel after preparation at 20 mm) to obtain the 1D swelling factor $SF_L = L/L_{dry}$, which is further used for the 1D actuation in the shape-morphing architecture.

Comparison of volume phase transition temperature of different thermoresponsive networks: The mTEGA/eDEGA copolymer network achieves a higher SF_L compared to the commonly used poly(*N*-isopropylacrylamide). The volume phase transition temperature of the copolymer network is not greatly affected by the introduction of Ca^{2+}-crosslinked alginate (**Figure S2**).

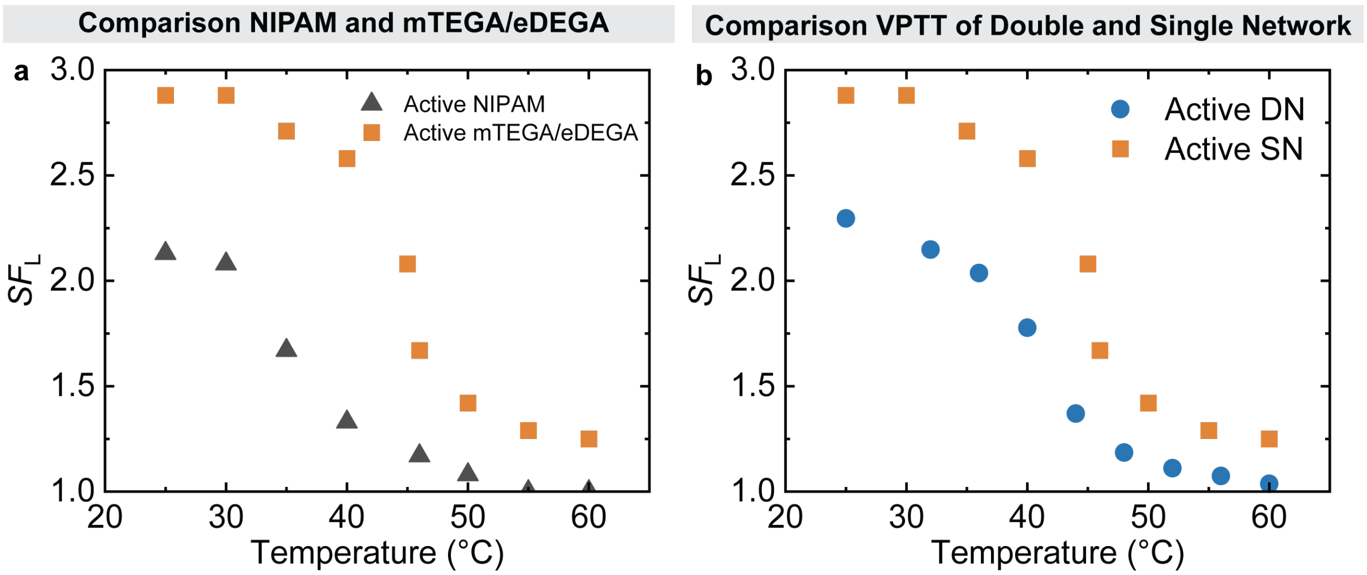

Figure S2: (**a**) Comparison of two thermoresponsive polymer networks. SF_L of the reference rod-like single network using *N*-isopropylacrylamide (NIPAM) as monomer (0.88 M NIPAM, 0.38 mol% poly(ethylene glycol) diacrylate M_n = 6 kDa) and mTEGA/eDEGA as comonomer (0.87 M mTEGA and 0.58 M eDEGA, 0.23 mol% poly(ethylene glycol) diacrylate M_n = 6 kDa). (**b**) Comparison of the SF_L of the reference rod-like double network (DN) active hydrogel specimen which is used in this study and a single network (SN) (0.87 M mTEGA and 0.58 M eDEGA, 0.23 mol% poly(ethylene glycol) diacrylate M_n = 6 kDa) after equilibration in water at different temperatures.

Finite element simulation: To predict and optimize the actuation of the shape-morphing architecture, FE simulations are conducted using the structural mechanics module of COMSOL Multiphysics 5.6. Experimentally determined swelling lengths are used as an input. Neo-Hookean material model with a hydrogel bulk Poisson's ratio of ν_{bulk} = 0.45 is employed, and the respective Young's moduli after equilibration at 25 °C ($E_{active, 25 °C}$ = 7.5 kPa and $E_{passive, 25 °C}$ = 75 kPa), measured with a Shimadzu compact tabletop testing machine EZTest using a 2 N load cell and strain rate of 10 mm/min, are employed. Note that the Young's modulus after equilibration at 42 °C remains $E_{active, 42 °C}$ = 7.5 kPa and increases to $E_{active, 60 °C}$ = 10.9 kPa at 60 °C. In simulation, the observed increase of the effective Young's modulus with increase of temperature (decrease of swelling factor) is associated with the change of the cross-section. Furthermore, the active hydrogel specimen swollen to equilibrium at 25 °C (SF_L = 2.26) reaches a contraction strength of 3.03 ± 0.04 kPa when exposed to hot water at 85 °C (**Figure S3b**).

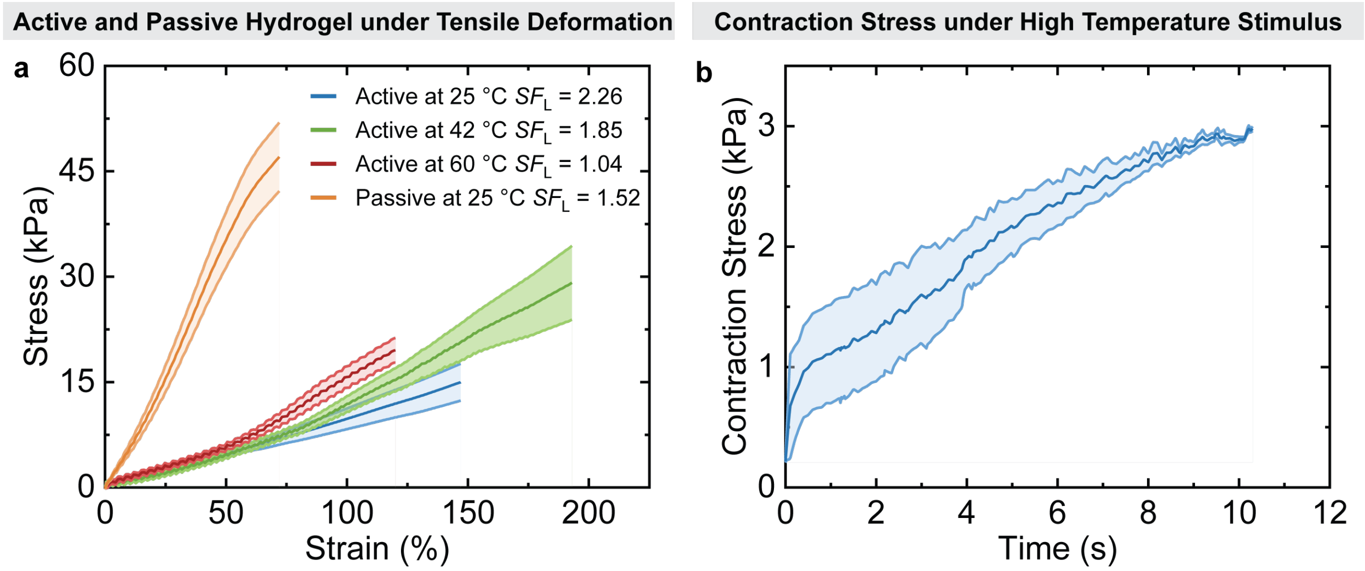

Figure S3: (**a**) Tensile testing curves of the active material after swelling to equilibrium at 25 °C (SF_L = 2.26), 42 °C (SF_L = 1.85) and 60 °C (SF_L = 1.04) as well as the passive material after swelling to equilibrium at 25 °C (SF_L = 1.52). The graphs represent the averaged curves with standard error of n = 12 specimens each. (**b**) Contraction stress under high temperature stimulus over time. The active hydrogel specimen swollen to equilibrium at 25 °C (SF_L = 2.26) is clamped inside the tensile testing set-up without deformation. The specimen is exposed to hot water at 85 °C and the contraction stress over time is recorded. The specimen reaches a contraction strength of 3.03 ± 0.04 kPa. The graph represents the averaged curve with standard error of n = 3 specimens.

Swelling is simulated using isotropic thermal expansion. All simulations described in the main text are performed in 2D plane stress settings for specimen with geometry identical to experimental. The boundary effects do not play a major role as shown in **Figure S6b**, comparing the employed model, 2D and 3D models of corresponding unit cells with superimposed periodic boundary conditions.

Determination of the Unit Cell Angle and Poisson's Ratio: The shape-morphing geometries are also measured ex-situ: The structural angle is measured with ImageJ after photographing (Panasonic DMC-G70KAEGK camera) the geometry under UV light (λ = 365 nm) to give higher contrast images of the fluorescing rhodamine B dye.

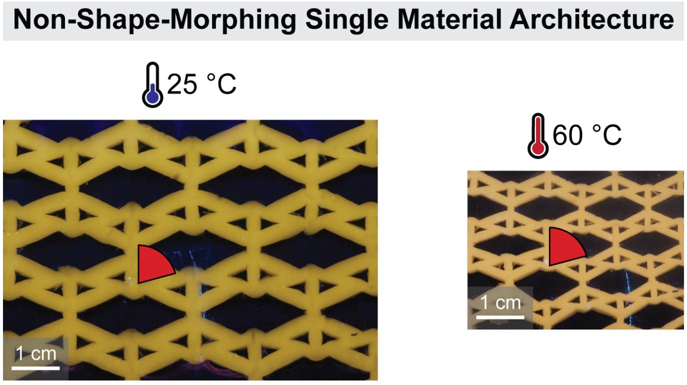

Figure S4: Non-shape-morphing single material architecture after equilibration at room temperature and 60 °C. The structural angle remains 75° as the whole structure scales isotropically.

The Poisson's ratio is determined from photographs where the dimension of the centered unit cell is measured at different elongations via $\nu = \frac{\varepsilon_{yy}}{\varepsilon_{xx}} = -\frac{v_{top}-v_{bottom}}{h} / \frac{u_{right}-u_{left}}{l}$. The shape-morphing architecture is gripped at three points on both sides then one side is moved in 1 mm steps. The deformation is increased until high tension is built up which would lead to structural rupture. Due to the slow re-swelling, hydrogel specimens can be removed from the water bath and imaged at room temperature under increasing deformation without significant re-swelling taking place.

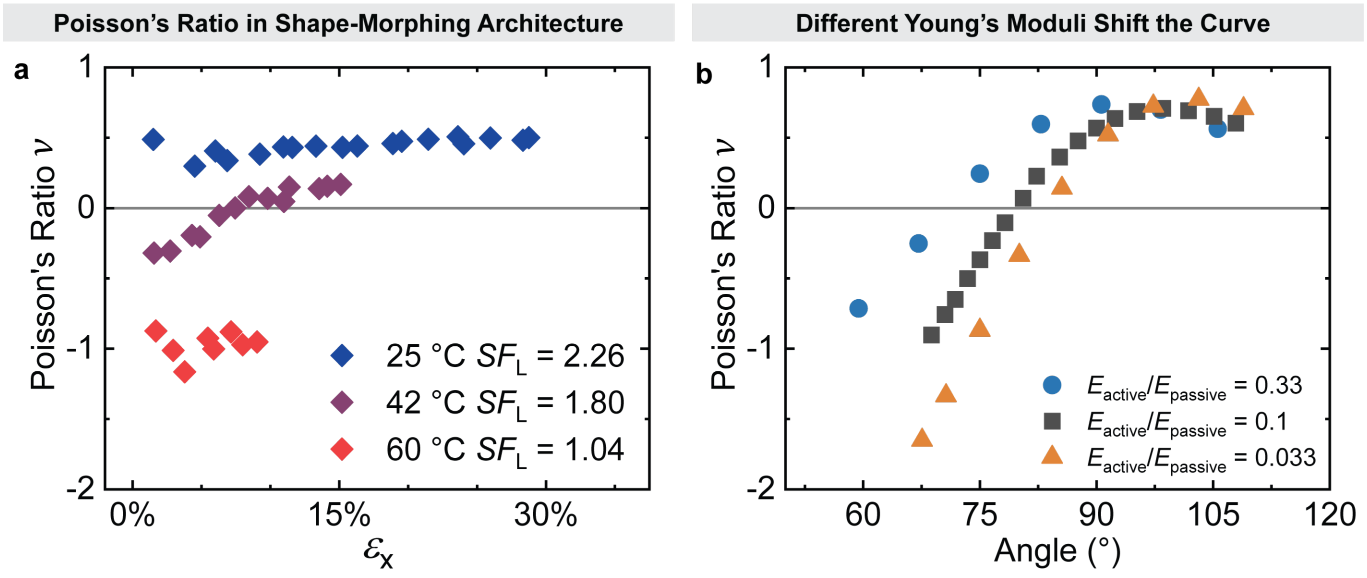

Figure S5: (**a**) Experimental Poisson's ratios versus strain for the shape-morphing dual material architecture. (**b**) Poisson's ratio at different starting angle where a decreasing ratio of $E_{active}/E_{passive}$ shifts the curve to higher angles.

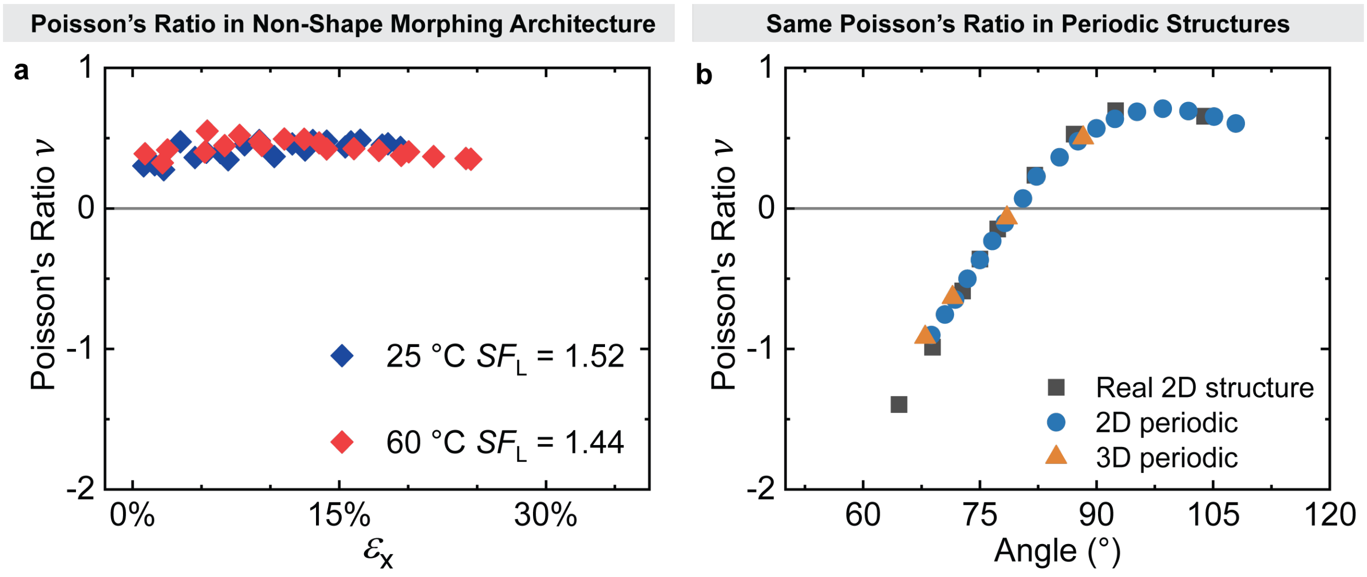

Figure S6: (**a**) Experimental Poisson's ratio versus strain for the non-shape morphing single material architecture at different swelling equilibria. The non-shape morphing architecture keeps a constant positive Poisson's ratio. (**b**) Simulation of the Poisson's ratio at different starting angle shows the same behavior in the full 2D specimen as compared to respective periodic 2D-geometry as well as periodic 3D-geometry with thickness 2 mm.
